# Automatic Calibration of a Two-Axis Rotary Table for 3D Scanning Purposes

**DOI:** 10.3390/s20247107

**Published:** 2020-12-11

**Authors:** Livio Bisogni, Ramtin Mollaiyan, Matteo Pettinari, Paolo Neri, Marco Gabiccini

**Affiliations:** Dipartimento di Ingegneria Civile e Industriale, Università di Pisa, Largo Lucio Lazzarino 1, 56122 Pisa, Italy; l.bisogni@studenti.unipi.it (L.B.); r.mollaiyan@studenti.unipi.it (R.M.); m.pettinari1@studenti.unipi.it (M.P.); marco.gabiccini@unipi.it (M.G.)

**Keywords:** rotary table calibration, 3D scanning, optical system

## Abstract

Rotary tables are often used to speed up the acquisition time during the 3D scanning of complex geometries. In order to avoid manual registration of the point clouds acquired with different orientations, automatic algorithms to compensate the rotation were developed. Alternatively, a proper calibration of the rotary axis with respect to the camera system is needed. Several methods are available in the literature, but they only consider a single-axis calibration. In this paper, a method for the simultaneous calibration of both axes of the table is proposed. A checkerboard is attached to the table, and several images with different poses are acquired. An optimization algorithm is then setup to determine the orientation and the locations of the two axes. A metric to assess the calibration quality was also defined by computing the average mean reprojection error. This metric is used to investigate the optimal number and distribution of the calibration poses, demonstrating that the optimum calibration results are achieved when a wider dispersion of the calibration poses is adopted.

## 1. Introduction

Image-based modeling is the process of reconstructing the three-dimensional geometry and the surface appearance of a real object from its two-dimensional images. In typical applications [1] images are acquired in a controlled environment. In order to achieve a $360{}^{{}^{\circ}}$ view of a complex object, the scanning must be repeated from different perspectives. Then, acquisition performed from different viewpoints needs to be registered, to reconstruct the full object geometry. The easiest solution to this issue is the manual point cloud registration, which is generally based on the 3-2-1 procedure: the first rough registration is manually provided by selecting three or more corresponding points on the clouds. Then, an iterative closest-point algorithm is run to refine the manual registration [2]. This procedure is robust and versatile, and it does not impose any constraints in terms of scanning perspective, but it requires long processing times. The procedure can be accelerated by gluing markers on the target surface, which can help the user to manually select the corresponding points, or can even allow for automatic algorithms [3]. Nevertheless, the marker placement may be cumbersome and also impossible to use where the surface cannot be altered. An alternative solution to this problem is represented by the use of turntables, which have proven to be effective in the reconstruction of complex shapes [4,5]. The object to be scanned can be placed on a turntable, and a stereo-camera system acquires images, while both orientation and placement of the item are changed within the work space by rotating the table by known angles. The use of a rotary table imposes a constraint to the possible scanning orientations, which can help the development of automatic algorithms to register the point clouds [6,7]. In addition, knowing the precise position and orientation of the turntable axis can further improve the registration step [8,9,10]. To this extent, a proper calibration of the acquisition system is necessary to obtain satisfying results.

The camera calibration problem has been extensively studied by researchers, and several approaches have been proposed. Linear methods [11,12] adopt a simple pinhole camera model and incorporate no distortion effects. Despite the algorithm being fast and non-interactive, neglecting camera distortion implies that lens distortion effects cannot be compensated for. Conversely, non-linear techniques [13,14,15] use general camera models, which also incorporate distortion parameters. At first, a relation between parameters is established, and then an iterative solution is found by minimizing properly defined error terms. Typically, the iterative procedure requires an initial guess, which often dictates the quality of the convergence. However, the most common approach is represented by two-step techniques [16,17,18], where in the first step, a direct solution of some parameters is given, and then an iterative procedure is followed in order to compute the other parameters as well as to reduce the error of the direct solution. The research on stereo camera systems calibration pushed forward the calibration of non-conventional optical setups based on mirrors (catadioptric systems), such as [19,20,21,22] which solve the problem of spherical mirror calibration to achieve an omnidirectional scanning.

Although technical literature well addresses the camera calibration problem, the extraction of the rotation axis of the turntable with reference to the fixed camera coordinate frame still deserves attention. Many different approaches have been found dealing with single-axis calibration: Mülayim et al. [23] proposed a multi-image approach capable of extracting the rotation axis of a single-axis turntable with respect to the camera coordinate frame. Li et al. [24] calibrated the rotation axis by means of a test sphere with a known diameter mounted on the turntable, similarly to [25]. Bi et al. [26] used a cuboid block and edge detection to assess the rotation axis. Langming et al. [27] developed a procedure based on the scanning of a cylindrical specimen. Ye et al. [28] used a planar specimen acquired in different orientations, basing the calibration on the intersection lines between subsequent planar surfaces. Chen et al. [29] formalized the problem as a constrained global optimization problem. However, these techniques do not generalize when a multi-axis turntable is considered, since each axis has to be calibrated separately, one after the other. Furthermore, no strict information about the placement of the calibration target as well as the number of images to be acquired is given in order to obtain acceptable results.

In this study, a novel approach for the calibration of a two-axis turntable is proposed. At first, a mathematical model for the rotation of a point attached to the turntable has been formalized and exploited in order to provide a minimum least-squares estimation of the two rotation axes as well as their distance with respect to the reference camera. Afterwards, a further analysis, based on optimal conditions to calibrate the rotary table, was conducted in order to optimize the estimates of the calibration process. Optimal conditions are considered to be the number and the orientation of placements of the calibration board with respect to the stereo-camera system. Qualitative criteria are finally provided based on experimental results.

## 2. Materials

**Figure 1:**
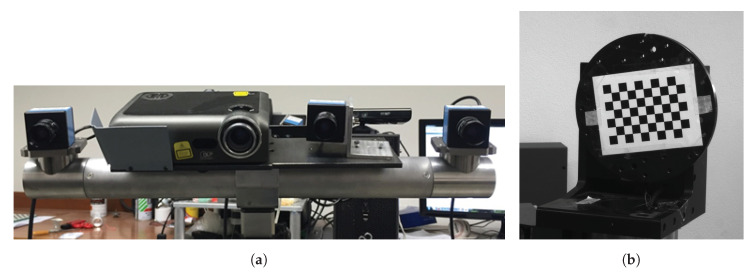
Main instruments used for experimentation: (**a**) stereo-camera system; (**b**) two-axis turntable with checkerboard attached.

**Figure 2:**
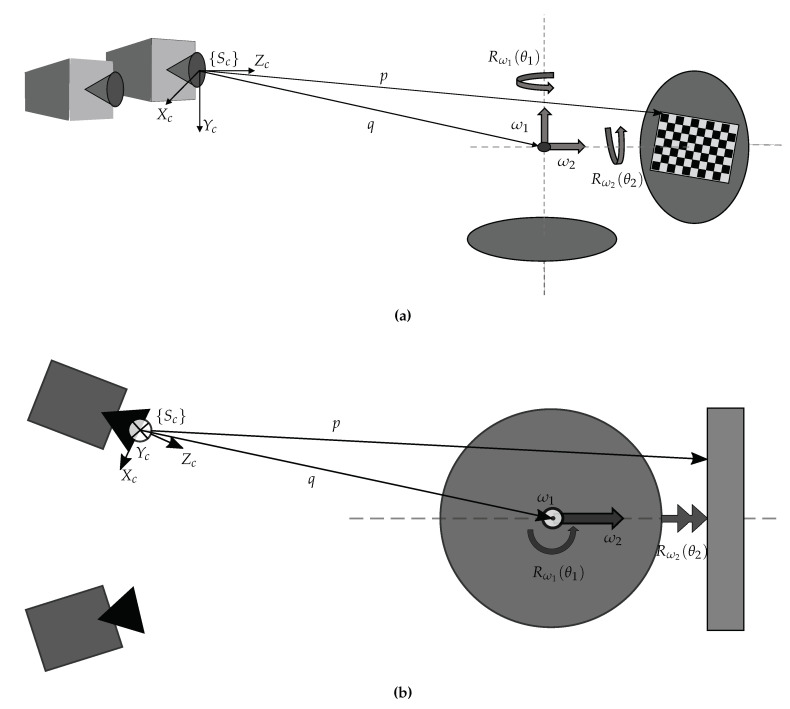
Axes and system representations: (**a**) 3D axonometric view; (**b**) 2D top view.

With reference to Figure 1 the image acquisition system consisted of a fixed stereo-camera system, a rotary table, and a computer. The stereo-camera system, shown in Figure 1a, is characterized by two digital cameras with a resolution of 1600 pixel × 1200 pixel (The ImagingSource® DMK 51BU02), equipped with lenses having a 16 mm focal length, and a 1024 pixel × 768 pixel multimedia DLP projector (OPTOMA EX330e, resolution XGA 1024 pixel × 768 pixel). Figure 1b shows the two-axis rotary table developed in this work. The overall system schematic, reporting the main reference frames, is represented in Figure 2. The first axis is parallel to the camera image plane while, by construction, the second is perpendicular to the first; the two axes are also assumed to intersect each other. It is reasonable to make such an assumption, since the rotary table was designed as a CNC (Computer Numerical Control) machine with high dimensional accuracy. An asymmetrical checkerboard of 7 × 10 squares (each square having size of 12 mm × 12 mm), resulting in M=54 inner corners, was attached to the turntable during the image acquisition process. This pattern allows for extraction of the point sets as they are placed in corners on the squares (excluding those on the external side of the checkerboard). Therefore, information on the table configuration, through the arrangement of the points in space, can then be deduced.

Each axis of the turntable was moved by means of a stepper motor, respectively a NEMA 23 for the vertical axis and a NEMA 17 for the horizontal axis. Both steppers were controlled via Arduino Uno with the addition of the Adafruit Motor Shield V2. The overall system was controlled via Matlab^®^ on a PC platform where a Graphic User Interface (GUI) has been developed in order to simplify the entire workflow, providing an interface that allows to control the settings of cameras, image acquisition, table calibration, and analysis results. The software is available as a free package at [30], and a brief description of the GUI is given in Appendix A.

## 3. Calibration Approach

The geometrical construction of the system was exploited for the implementation of a suitable calibration procedure of the two-axis turntable. We assume that the centers of the tables are disposed such that their axes intersect perpendicularly at point *q*. That bears the advantage to consider a single point of application of rotation for both axes. Hence, just a single position vector from the cameras is enough. Since the orientation of the camera system, chosen as a global reference, was different from those of the tables, axis-angle notation was used to describe the rotation of each table.

**Figure 3:**
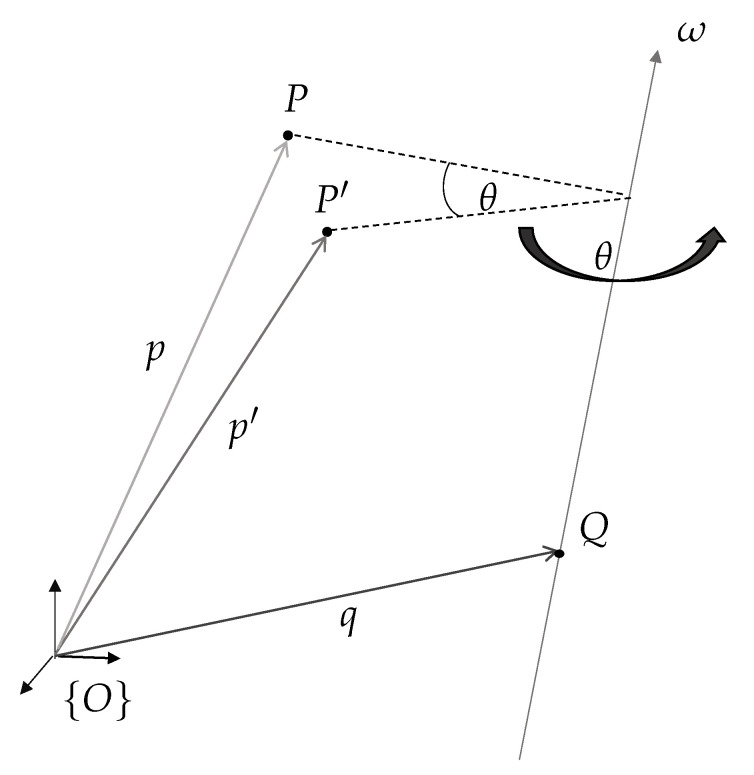
Rotation of a point *p* by an angle θ around a single axis ω.

The general rotation of a point around an axis outside of the origin, as shown in Figure 3, is represented as follows:

(1) $$p^{\prime }=R_{\omega }\left(\theta \right)\left(p-q\right)+q,$$

where *p* is a generic point positioned on a system rotating around an axis with unit vector ω going through point *q*, $p^{\prime }$ is the rotated point, and $R_{\omega }\left(\theta \right)$ is the axis-angle parametrization of the rotation matrix in which θ is the angle of rotation. Equation (1) can be expressed as a rigid-body transformation in homogeneous coordinates as follows:

(2) $$\left[\begin{array}{c} p^{\prime }\\ 1 \end{array}\right]=\left[\begin{array}{cc} R_{\omega }\left(\theta \right) & (I-R_{\omega }\left(\theta \right))q\\ 0 & 1 \end{array}\right]\left[\begin{array}{c} p\\ 1 \end{array}\right]$$

Equation (2) can be applied to the studied system. As shown in Figure 2, the axis of the horizontal table is defined as $\omega_{1}$, the axis of the vertical as $\omega_{2}$, $\theta_{1}$ is the angle of rotation around $\omega_{1}$, and $\theta_{2}$ the angle around $\omega_{2}$. Since $\omega_{2}$ lies in the reference system of the horizontal table, the global transformation must be defined in the following order:

(3) $$\begin{array}{cc} \left[\begin{array}{c} p^{\prime }\\ 1 \end{array}\right] & =T\left(\omega_{1},\omega_{2},q;\left(\theta_{1},\theta_{2}\right)\right)\left[\begin{array}{c} p\\ 1 \end{array}\right]\\ \; & =\left[\begin{array}{cc} R_{\omega_{1}}\left(\theta_{1}\right) & (I-R_{\omega_{1}}\left(\theta_{1}\right))q\\ 0 & 1 \end{array}\right]\left[\begin{array}{cc} R_{\omega_{2}}\left(\theta_{2}\right) & (I-R_{\omega_{2}}\left(\theta_{2}\right))q\\ 0 & 1 \end{array}\right]\left[\begin{array}{c} p\\ 1 \end{array}\right]\\ \; & =\left[\begin{array}{cc} R_{\omega_{1}}\left(\theta_{1}\right)R_{\omega_{2}}\left(\theta_{2}\right) & (I-R_{\omega_{1}}\left(\theta_{1}\right)R_{\omega_{2}}\left(\theta_{2}\right))q\\ 0 & 1 \end{array}\right]\left[\begin{array}{c} p\\ 1 \end{array}\right] \end{array}$$

The axis-angle representation provides the advantage of being a direct function of the components of the axis unit vector for both tables. The two axis unit vectors $\omega_{1}$ and $\omega_{2}$, together with the global coordinates of their intersection point *q* from the camera frame, denoted in Figure 2 as $\left\{S_{c}\right\}$, are used as input parameters for an optimization procedure that finds the solutions through a nonlinear least-squares approach.

First, an initial reference pose $X^{R}$ is acquired, which is associated to angle configuration $\left(\theta_{1}=\theta_{2}\right)=\left(0{}^{{}^{\circ}},0{}^{{}^{\circ}}\right)$. Then, *N* poses ${\bar{X}}^{i}$ (i=1,…,N) are acquired by rotating around both axes $\omega_{1}$ and $\omega_{2}$ in their working range of interest. From each of the *N* poses, a point cloud in the 3D space is extracted, corresponding to the checkerboard corners, thus obtaining the measured pose ${\bar{X}}^{i}$. The theoretical relation between a generic (ideal) pose $X^{i}$ and the reference pose $X^{R}$ would be

(4) $$X^{i}=T\left(\omega_{1},\omega_{2},q;{\left(\theta_{1},\theta_{2}\right)}_{i}\right)X^{R},\;i=1,\ldots ,N$$

where both $X^{i}$ and $X^{R}$ are expressed in homogeneous coordinates. However, since *measured* point clouds ${\bar{X}}^{i}$ are considered, which are affected by various error sources, the following equations can be written:

(5) $$\left\{\begin{array}{c} \begin{array}{cc} {\bar{X}}^{1}-T\left(\omega_{1},\omega_{2},q;{\left(\theta_{1},\theta_{2}\right)}_{1}\right)X^{R} & =\delta X^{1}\\ {\bar{X}}^{2}-T\left(\omega_{1},\omega_{2},q;{\left(\theta_{1},\theta_{2}\right)}_{2}\right)X^{R} & =\delta X^{2}\\ \; & =\\ {\bar{X}}^{N}-T\left(\omega_{1},\omega_{2},q;{\left(\theta_{1},\theta_{2}\right)}_{N}\right)X^{R} & =\delta X^{N} \end{array} \end{array}\right.$$

where $\delta X^{i}\in R^{4\times M}$ (i=1,…,N) are the residual errors between measured and ideal coordinates of corresponding point clouds originated by $X^{R}$, and where *M* are the points (extracted via software) on the checkerboard.

Finally, a nonlinear least-squares estimation of the parameters is established to minimize the mismatch between measured and ideal point cloud coordinates. The optimization function is therefore based on Equation (5), where the table rotation angles are known by counting the steps provided to the stepper motors (since the rotational speed is low, it is reasonable to neglect step losses). On the other hand, $q,\omega_{1},\;\mathrm{and}\;\omega_{2}$ are unknown and should be estimated. A formalization of the nonlinear least-squares estimation, minimizing the sum of squared Euclidean error norms, is provided as follows:

(6) $$\min_{\omega_{1},\omega_{2},q}\;\;\frac{1}{2}\sum_{i=1}^{N}\sum_{j=1}^{M}{∥{\bar{p}}_{j}^{i}-p_{j}^{i}\left(\omega_{1},\omega_{2},q;{\left(\theta_{1},\theta_{2}\right)}_{i}\right)∥}_{2}^{2},$$

where *i* is the pose index examined (i=1,…,N), *j* is the point index in the checkerboard (j=1,…,M), and ${\bar{p}}_{j}^{i}$ is the corresponding rotated point (Cartesian coordinates) measured on the checkerboard. Point $p_{j}^{i}\left(\omega_{1},\omega_{2},q;{\left(\theta_{1},\theta_{2}\right)}_{i}\right)$ is obtained from the rotation of the reference pose by $\theta_{1,i}$ around axis $\omega_{1}$ and $\theta_{2,i}$ around axis $\omega_{2}$, both intersecting at the point *q*. The optimization must also satisfy the following constraints:

{(7a)ω1Tω2=0(7b)ω1Tω1=1(7c)ω2Tω2=1

where (7a) indicates the perpendicularity between the axes, whereas (7b) and (7c) impose the condition for $\omega_{1},\omega_{2}$ of having unit length.

**Figure 4:**
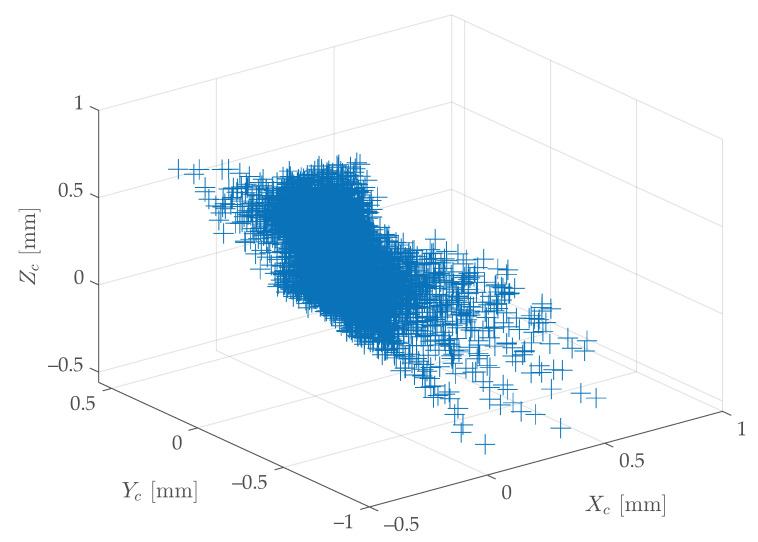
Dispersion of the M=54 point-to-point errors in the 3D space—for a given set of N=100 images—committed using an estimation of $\left(\omega_{1},\omega_{2},q\right)$ yielded by (6) and expressed in the camera frame $\left\{S_{c}\right\}$.

**Figure 5:**
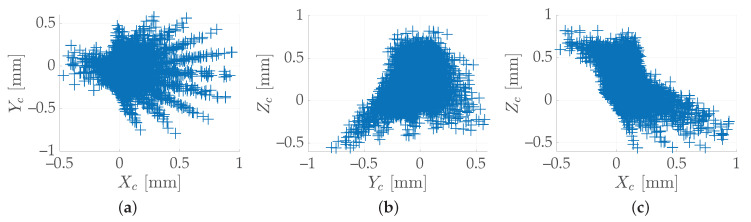
Same error cloud of Figure 4, plotted in the three Cartesian planes: (**a**) XY-plane; (**b**) YZ-plane; (**c**) ZX-plane.

For instance, the proposed method was employed for the calibration of a two-axis rotary table, using the hardware previously described in Section 2; hence, M=54 points per grid were considered. At first, 100 stereo-pair images were acquired in different poses ${\left(\theta_{1},\theta_{2}\right)}_{i},i=1,\ldots ,N$ of the rotary table. Then, using an estimation of $\left(\omega_{1},\omega_{2},q\right)$ yielded by (6) applied to all the N=100 stereo-pair images, and by taking the inverse of (4), the measured grids ${\bar{X}}^{i},i=1,\ldots ,N$ were reprojected onto the reference grid ${\bar{X}}^{R}$. Finally, the N·M point-to-point errors committed were computed. Figure 4 shows the error cloud in the 3D space, expressed in the camera frame $\left\{S_{c}\right\}$, whereas Figure 5 shows these errors in the three Cartesian planes. The absolute error was lower than 1 mm in every direction. 

## 4. Influence of the Number of Calibration Images

Calibration quality depends both on the number *K* of poses used and on their spatial location, i.e., ${\left(\theta_{1},\theta_{2}\right)}_{i}$, i=1,…,K. Hence, it is useful to investigate the influence of the number *K* of images to be captured towards a quality and fast calibration. To this extent, a proper metric was introduced, as discussed further in this section.

### 4.1. Data Set Definition

Any *i*-th image, i=1,…,K, can be captured in the rotary table *joint configuration Space* *S*, $S\subseteq \left[-180^{{}^{\circ}},180^{{}^{\circ}}\right]\times \left[-180^{{}^{\circ}},180^{{}^{\circ}}\right]$. This is the space any pair of angular positions ${\left(\theta_{1},\theta_{2}\right)}_{i}$, i=1,…,|S|, belongs to, i.e., $S=\left[\theta_{1,min},\theta_{1,max}\right]\times \left[\theta_{2,min},\theta_{2,max}\right]$. In order to assess the influence of the number of images *K*, a test run was conducted. First of all, a set of images was captured in *S* (*Acquisition set* *A*, A⊂S). Then—excluding an image $S_{R}$ which is taken as Reference—it was partitioned into two subsets: the *Calibration set* *C*, C⊂A, and the *Test set* *T*, T⊂A, so that $C\cup T=A\setminus S_{R}\;\wedge \;C\cap T=\emptyset$. *C* is the set from which to select the desired subset $C_{H}$ of *K* images to calibrate the turntable with, whereas *T* is the set subsequently used for quantifying the calibration error achieved with $C_{H}$. With respect to *C*, for each *K*, K=1,…,|C|, a series of $b_{K}$ subsets $C_{H}$, $C_{H}\subseteq C$ could be extracted, where *H* is a set of indices *i*, *K* is the size of *H*, and $b_{K}$ is the following binomial coefficient:

(8) bK=|C|K=|C|!K!(|C|−K)!,K=1,…,|C|

**Figure 6:**
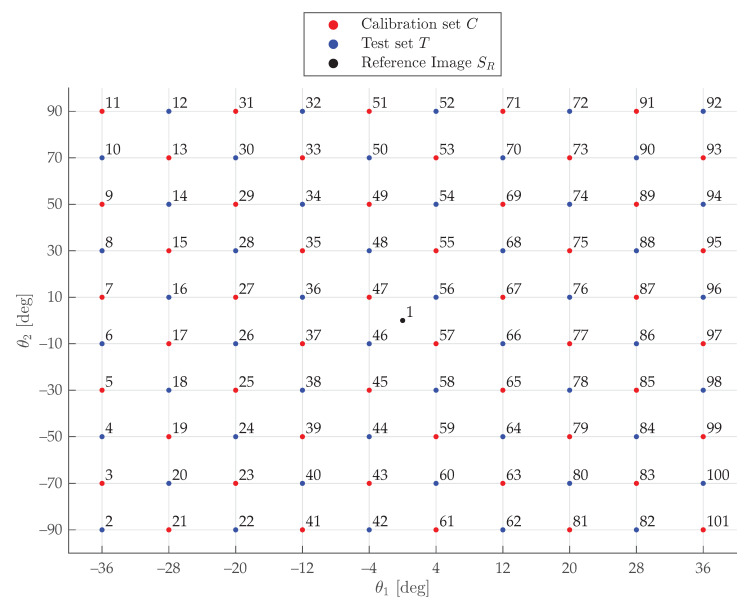
*Acquisition setA* within the *joint configuration Space* *S*; the red points constitute the *Calibration set* *C*, whereas the blue are the *Test set* *T* and black is the reference. The number next to a point of coordinates ${\left(\theta_{1},\theta_{2}\right)}_{i}$ denotes the index *i* of the corresponding image; any combination of the indices highlighted in red constitutes a possible subset *H* of size *K*.

In the present study, a $S=\left[-36{}^{{}^{\circ}},+36{}^{{}^{\circ}}\right]\times \left[-90{}^{{}^{\circ}},+90{}^{{}^{\circ}}\right]$ was considered. Such *S* ensures that the checkerboard remains visible from the stereo-camera system during the entire acquisition process. To discretize *S*, a uniform sampling was performed, obtaining 101 images: the first one was associated to $\left(0{}^{{}^{\circ}},0{}^{{}^{\circ}}\right)$ and was taken as reference, whereas the remaining 100 were equally spaced in steps of size $\left(\delta \theta_{1},\delta \theta_{2}\right)=\left(8^{{}^{\circ}},20^{{}^{\circ}}\right)$ within *S*, as in Figure 6. Lastly, the obtained *A* was partitioned into two equally sized and spaced sets *C* and *T*. In more detail:

- $S=\left[\theta_{1,min},\theta_{1,max}\right]\times \left[\theta_{2,min},\theta_{2,max}\right]=\left[-36^{{}^{\circ}},+36^{{}^{\circ}}\right]\times \left[-90^{{}^{\circ}},-90^{{}^{\circ}}\right]$;
- $A=\{{\left(\theta_{1},\theta_{2}\right)}_{i}|i=1,\ldots ,N_{A}\}$, where $N_{A}=\left|A\right|=101$;
- $C=\{{\left(\theta_{1},\theta_{2}\right)}_{2r+1}|r=1,\ldots ,N_{C}\}$, where $N_{C}=\left|C\right|=\frac{N_{A}-1}{2}=50$;
- $T=\{{\left(\theta_{1},\theta_{2}\right)}_{2s}|s=1,\ldots ,N_{T}\}$, where $N_{T}=\left|T\right|=\frac{N_{A}-1}{2}=50$;
- $H\subseteq \{3,\ldots ,2r+1,\ldots ,N_{A}\}$ is a set of indices *i*, where $r=1,\ldots ,N_{C}$, that denotes the pairs of angular positions ${\left(\theta_{1},\theta_{2}\right)}_{i}$, i=2r+1, of the subset of images to calibrate with;
- K=|H|, $K\in \{1,\ldots ,N_{C}\}$, is the size of *H*, i.e., the number of images considered;
- $C_{H}=\left\{{\left(\theta_{1},\theta_{2}\right)}_{h}|h\in H\right\}$.

The *i*-th pair of angular positions is given by

(9) $$\begin{array}{c} {\left(\theta_{1},\theta_{2}\right)}_{i}=\left\{\begin{array}{cc} (0{}^{{}^{\circ}},0{}^{{}^{\circ}})\; & \;i=1\\ (\theta_{1,min}+\left(m-1\right)\;\cdot \;8{}^{{}^{\circ}},\theta_{2,min}+\left(n-1\right)\;\cdot \;20{}^{{}^{\circ}})\; & \;i=2,\ldots ,N_{A} \end{array}\right. \end{array}$$

where

(10) $$\left\{\begin{array}{c} m=\left\lfloor \frac{i+8}{10}\right\rfloor \\ n=m\;\mathrm{mod}\;2\;\cdot \;\left(i+9-10m\right)+\left(m+1\right)\;\mathrm{mod}\;2\;\cdot \;\left(10m+2-i\right) \end{array}\right.\;i=2,\ldots ,N_{A}$$

### 4.2. Error Metric Definition

**Figure 7:**
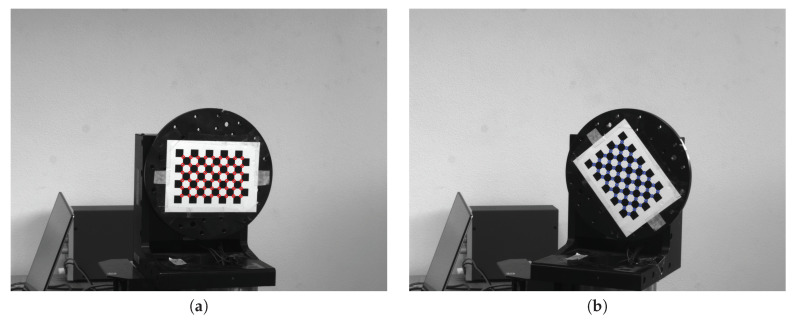
Checkerboard in two different poses and their *M* highlighted inner corners: (**a**) reference grid (${\bar{X}}^{R}$, in red); (**b**) target grid (${\bar{X}}^{i}$, in blue).

**Figure 8:**
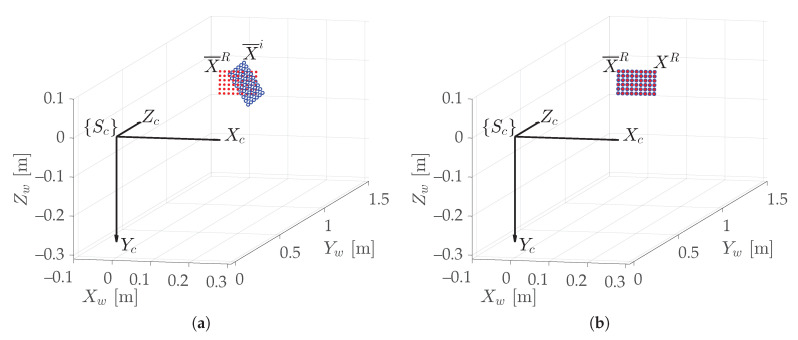
The two measured grids of Figure 7 in the operative space: (**a**) reference grid (${\bar{X}}^{R}$, in red; as in Figure 7a) and target grid (${\bar{X}}^{i}$, in blue; as in Figure 7b); (**b**) reference grid (in red), and target grid rotated by ${\left(\theta_{1},\theta_{2}\right)}_{i}$ onto the reference grid ($X^{R}$, in blue); virtually identical because of the low reprojection error.

The checkerboard, shown in Figure 7, consists of 7 × 10 squares, so the number of inner-corners *M* is (7−1)×(10−1)=54. Figure 7 shows the turntable with the attached checkerboard in two different poses: Figure 7a is the reference pose at ${\left(\theta_{1},\theta_{2}\right)}_{1}=\left(0{}^{{}^{\circ}},0{}^{{}^{\circ}}\right)$, whereas Figure 7b represents a generic *i*-th configuration, which is taken as target at ${\left(\theta_{1},\theta_{2}\right)}_{i}$. Given the *i*-th pose expressed in the coordinate frame $\left\{S_{c}\right\}$, $i=1,\ldots ,N_{A}$, and recognizing the *i*-th Equation in (5), it is possible to rotate by ${\left(\theta_{1},\theta_{2}\right)}_{i}$ its measured target grid ${\bar{X}}^{i}$ (blue dots in Figure 7b and Figure 8a) onto the reference grid ${\bar{X}}^{R}={\bar{X}}^{1}$ (red dots in Figure 7a and Figure 8), thus obtaining the reprojected grid $X^{R}=X^{1}$, as shown in Figure 8b.

Hence, given a subset $C_{H}$ within the *Calibration set* *C* of *K* images, and based on the results described in the previous section, it is possible to calibrate the rotary table, obtaining an estimate ${\left({\tilde{\omega }}_{1},{\tilde{\omega }}_{2},\tilde{q}\right)}_{H}$ of $\left(\omega_{1},\omega_{2},q\right)$. A series of metrics was arranged to quantify the reprojection accuracy obtained to reproject all $N_{T}$ grids of the *Test set* *T* onto the reference grid ${\bar{X}}^{1}$ using the calibration results acquired with the subset $C_{H}$ of *K* images. The subscript *H* highlights the dependency of the two axes, $\omega_{1}$ and $\omega_{2}$, and of their intersection point, *q*, on the particular subset of images used, thus ${\left({\tilde{\omega }}_{1},{\tilde{\omega }}_{2},\tilde{q}\right)}_{H}$.

Considering only the grids belonging to *T*, for a given *H*, the *point-to-point error* between a *j*-th measured point ${\bar{p}}_{j}^{1}$ on the reference grid ${\bar{X}}^{1}$ and the reprojection of the *j*-th point $p_{j}^{1}\left({\left({\tilde{\omega }}_{1},{\tilde{\omega }}_{2},\tilde{q}\right)}_{H};{\left(\theta_{1},\theta_{2}\right)}_{2s}\right)$ of the 2s-th target grid ${\bar{X}}^{2s}$ onto ${\bar{X}}^{1}$, i.e., $X^{1}=T^{-1}({\left({\tilde{\omega }}_{1},{\tilde{\omega }}_{2},\tilde{q}\right)}_{H};$${\left(\theta_{1},\theta_{2}\right)}_{2s}){\bar{X}}^{2s}$, where j=1,…,M and $s=1,\ldots ,N_{T}$, is

(11) $$\begin{array}{cc} \epsilon_{j,2s,H}={∥{\bar{p}}_{j}^{1}-p_{j}^{1}\left({\left({\tilde{\omega }}_{1},{\tilde{\omega }}_{2},\tilde{q}\right)}_{H};{\left(\theta_{1},\theta_{2}\right)}_{2s}\right)∥}_{2{\;}_{,}}\;\; & j=1,\ldots ,M\;,\;s=1,\ldots ,N_{T}\;,\;\\ \; & H\subseteq \left\{3,\ldots ,2r+1,\ldots ,N_{A}\right\}\;,\;r=1,\ldots ,N_{C} \end{array}$$

The arithmetic mean of $\epsilon_{j,2s,H}$ over *M* points yields the 2s-th *averaged point-to-point error* committed using the ${\left({\tilde{\omega }}_{1},{\tilde{\omega }}_{2},\tilde{q}\right)}_{H}$ that was estimated through the calibration previously made with the grids ${\bar{X}}^{h}$, $h\in H|{\left(\theta_{1},\theta_{2}\right)}_{h}\in C_{H}$, and for the target grid ${\bar{X}}^{2s}$, $s=1,\ldots ,N_{T}$:

(12) $$\epsilon_{2s,H}=\frac{\sum_{j=1}^{M}\epsilon_{j,2s,H}}{M},\;s=1,\ldots ,N_{T}\;,\;H\subseteq \left\{3,\ldots ,2r+1,\ldots ,N_{A}\right\}\;,\;r=1,\ldots ,N_{C}$$

Finally, averaging all of the $N_{T}$ *averaged point-to-point errors* over the entire *Test set* *T* gives the *T-averaged point-to-point error*, yielding the reprojection accuracy:

(13) $${\bar{\epsilon }}_{H}=\frac{\sum_{s=1}^{N_{T}}\epsilon_{2s,H}}{N_{T}},\;H\subseteq \left\{3,\ldots ,2r+1,\ldots ,N_{A}\right\}\;,\;r=1,\ldots ,N_{C}$$

Additionally, the root mean square (RMS) of these errors was defined as

(14) $${\bar{\sigma }}_{H}=\sqrt{\frac{\sum_{s=1}^{N_{T}}{\left(\epsilon_{2s,H}-{\bar{\epsilon }}_{H}\right)}^{2}}{N_{T}-1}},\;H\subseteq \left\{3,\ldots ,2r+1,\ldots ,N_{A}\right\}\;,\;r=1,\ldots ,N_{C}$$

The number of possible subsets $C_{H}$ of size *K* that can be employed for calibration is given by the binomial coefficient $b_{K}$ in (8). First of all, in order to have a rough estimation of the effect of *K* on the calibration accuracy, and to reduce the computational time, for each *K*, $K=2,\ldots ,N_{C}$ only $N_{R_{K}}=1000$ combinations of $C_{H}$ are randomly extracted, obtaining $N_{C}-1$ sets $R_{K}$, and $N_{R_{K}}$ is the size of $R_{K}$, i.e., $N_{R_{K}}=\left|R_{K}\right|$. The case K=1 is not considered from now on since it is not sufficient to calibrate a two-axis rotary table. Then, all the $R_{K}$ sets, $K=2,\ldots ,N_{C}$, were used to execute $\left(N_{C}-1\right)\;\cdot \;N_{R_{K}}=49,000$ independent table calibrations. Subsequently, their respective *T-averaged point-to-point errors* ${\bar{\epsilon }}_{H}$ given by Equation (13), $H|C_{H}\in R_{K}$, were computed. Finally, averaging them over $R_{K}$, where $K=2,\ldots ,N_{C}$, returns $N_{C}-1$*$R_{K}$-averaged point-to-point errors*:

(15) $${\bar{E}}_{K}=\frac{\sum_{H|C_{H}\in R_{K}}{\bar{\epsilon }}_{H}}{N_{R_{K}}},\;K=2,\ldots ,N_{C}$$

Additionally, the root mean square (RMS) of these errors was defined as

(16) $${\bar{\sigma }}_{K}=\sqrt{\frac{\sum_{H|C_{H}\in R_{K}}{\left({\bar{\epsilon }}_{H}-{\bar{E}}_{K}\right)}^{2}}{N_{R_{K}}-1}},\;K=2,\ldots ,N_{C}$$

**Figure 9:**
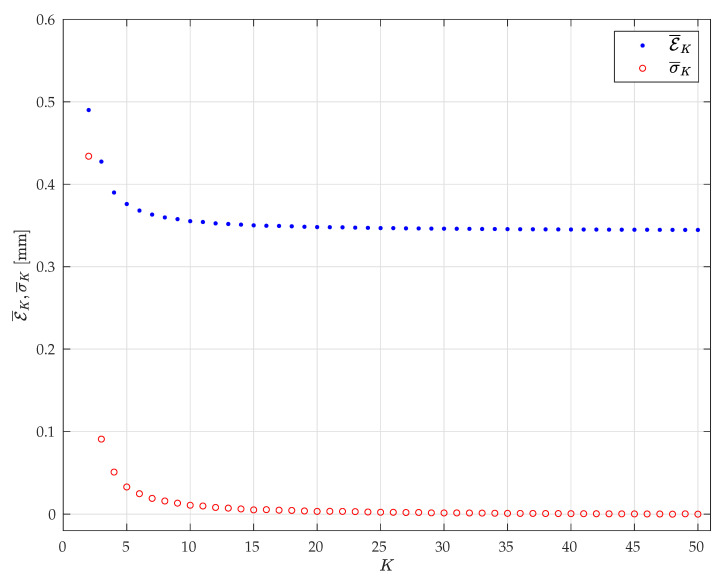
The *$R_{K}$-averaged point-to-point errors* ${\bar{E}}_{K}$ (blue dots) and their RMS ${\bar{\sigma }}_{K}$ (red circles) for $K=2,\ldots ,N_{C}$.

An overview of the errors, and their RMS, is presented in Figure 9 for $K=2,\ldots ,N_{c}$. As can be seen, both ${\bar{E}}_{K}$ and ${\bar{\sigma }}_{K}$ decrease when *K* increases. While the effect of *K* on ${\bar{E}}_{K}$ seems to saturate for *K* greater than 10, it can be noted that ${\bar{E}}_{K}$ still decreases with *K*. Thus, if the poses are randomly chosen, greater values of *K* are more likely to produce high-quality calibrations. Nevertheless, even a small *K* can provide high-quality calibration results, if the poses are properly chosen as demonstrated in the following.

### 4.3. Methods Comparison

In order to verify its effectiveness, the developed algorithm was compared with the circles method proposed by Chen et al. [29]. Hence, several combinations of images were extracted from the *Calibration Set*, and the calibration of the two-axis rotary table was carried out using both methods. It is worth mentioning, being that the circles method is a single-axis procedure, the vertical axis $\omega_{1}$ was firstly calibrated, and subsequently the horizontal axis $\omega_{2}$ was considered. For each combination *H* of images, the *T-averaged point-to-point error* ${\bar{\epsilon }}_{H}$ and its standard deviation ${\bar{\sigma }}_{H}$ were computed by considering all the images of the *Test Set*. The results are reported in Table 1, comparing the two methods for the tested calibration sets.

It is possible to observe that the proposed method returned almost constant values of ${\bar{\epsilon }}_{H}$ and ${\bar{\sigma }}_{H}$ (i.e., 0.33–0.36 mm and 0.16–0.18 mm, respectively), which slightly decreases with the increasing of *K*. On the contrary, a different behavior was noticed in the circles procedure case, where ${\bar{\epsilon }}_{H}$ decreased but not ${\bar{\sigma }}_{H}$, since this method is influenced appreciably by the specific calibration poses. In addition, the range of values of both ${\bar{\epsilon }}_{H}$ was considerably wider (i.e., 0.52–1.70 mm) than the results of the proposed method. Hence, the proposed method seems to be more robust and reliable with respect to the circles algorithm.

## 5. Optimal Calibration Set

**Figure 10:**
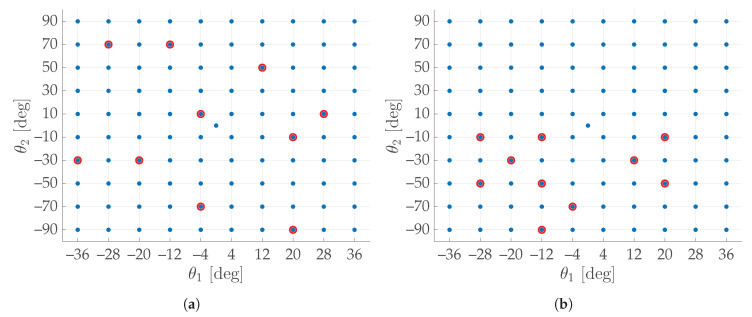
Two investigated subsets with the same number *K* of points ${\left(\theta_{1},\theta_{2}\right)}_{h}$ within the *joint configuration Space S*: (**a**) *H* = {5, 13, 25, 33, 43, 47, 69, 77, 81, 87}, *K* = 10, *C_H_* = {(−36°,−30°), (−28°, 70°), (−20°,−30°), (−12°, 70°), (−4°,−70°), (−180°, 10°), (12°, 50°), (20°,−10°), (20°,−70°), (28°, 10°)}, which has a higher dispersion; (**b**) *H* = {17, 19, 25, 37, 39, 41, 43, 65, 67, 79}, *K* = 10, *C_H_* = {(−28°,−10°), (−28°,−50°), (−20°,−30°), (−12°,−10°), (−12°,−50°), (−12°,−90°), (−4°,−70°), (12°,−30°), (20°,−10°), (20°,−50°)}, which has a lower dispersion.

On the basis of the data obtained in the previous section, a visual inspection was conducted to find out whether a certain pattern in the *joint configuration Space* *S* yielded preferred calibration results. No recurrent pattern (e.g., “X”-like, cross-like, circle-like, square-like, etc.) was evident among either of the studied subsets, which had led to quality calibrations of the tables or among the ones with poorer calibrations. Nevertheless, a direct correlation between dispersion of subsets and calibration quality has been found: for any given *H*, the more uniformly distributed the cloud of *K* points $(\theta_{1}$, $\theta_{2}{)}_{h}\in C_{H}\subset S$, h∈H, the lower its calibration error ${\bar{\epsilon }}_{H}$. For instance, in Figure 10, two opposite situations are reported. A wide dispersion can be noted in (a), corresponding to ${\bar{\epsilon }}_{H}=0.3445$ mm, while a lower dispersion can be noted in (b), which corresponds to ${\bar{\epsilon }}_{H}=0.4239$ mm.

### 5.1. Complete Graph Index

**Figure 11:**
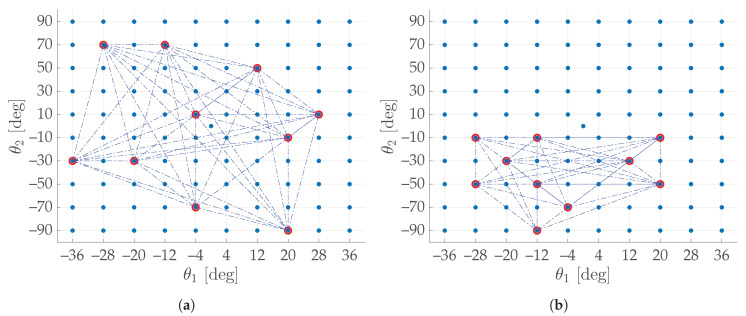
Complete graphs on the nodes in *C_H_* of Figure 10: the *K* red points are the vertices, whereas the $\left(\begin{array}{c} K\\ 2 \end{array}\right)$ blue dotted lines constitute the edges: (**a**) *C_H_* is the same as Figure 10a; (**b**) *C_H_* is the same as Figure 10b.

To quantify the scattering of any desired set of arbitrary dimension *K*, an index ($I_{CG}$) was devised based on the calculations of the arithmetic mean of all the weights of a weighted complete graph (*CG*). The subset of Figure 10a can be considered as an example. If its points were regarded as the vertices of a graph, it would be possible to think of their edges as being weighted by their Euclidean distances; hence, they can be summed and finally averaged by the number of links. Among all the weighted undirected graphs, the weighted complete graph (*CG*) was chosen, since it has the property to take into account all the pairs of points. In Figure 11 the *CG* constructed from the subset of Figure 10a is shown; it is possible to see its *K* points (nodes of *CG*, in red) and their $\left(\begin{array}{c} K\\ 2 \end{array}\right)$ links (edges of *CG*, in blue).

Given *K* points $v_{t}={\left(\theta_{1},\theta_{2}\right)}_{t},\;t=1,\ldots ,K$, it is advantageous to normalize them so that the index does not depend on the length of *S* ($\left[\theta_{1,min},\theta_{1,max}\right]\times \left[\theta_{2,min},\theta_{2,max}\right]$). The normalized angular positions ${\hat{v}}_{t}=({\hat{\theta }}_{1}$, ${\hat{\theta }}_{2}{)}_{t},\;t=1,\ldots ,K$, are obtained by applying the following normalizations:

(17) $${\left({\hat{\theta }}_{1},{\hat{\theta }}_{2}\right)}_{t}=\frac{{\left(\theta_{1},\theta_{2}\right)}_{t}-\left(\theta_{1,min},\theta_{2,min}\right)}{\left(\theta_{1,max},\theta_{2,max}\right)-\left(\theta_{1,min},\theta_{2,min}\right)},\;t=1,\ldots ,K$$

where ${\left({\hat{\theta }}_{1},{\hat{\theta }}_{2}\right)}_{t}$ are the normalized coordinates of ${\hat{v}}_{t}$ along the $\omega_{1}$-axis and the $\omega_{2}$-axis, respectively. The weighted complete graph of order *K* considered is the pair (G,w), where

- $V=\{{\hat{v}}_{t}|t=1,\ldots ,K\}$ is the *set of vertices* (normalized angular positions);
- $E=\{\left({\hat{v}}_{t},{\hat{v}}_{u}\right)|t,u=1,\ldots ,K,\;u>t\}$ is the *set of edges*;
- G=(V,E) is a graph;
- $\left|E\right|=K2=\frac{K(K-1)}{2}$;
- $w:E\to R_{\geq 0}$ is the *weight function*;

and the *weight function* is the Euclidean distance between any pair of vertices:

(18) $$w={∥{\hat{v}}_{t}-{\hat{v}}_{u}∥}_{2}={\sqrt{{\left({\hat{\theta }}_{1,t}-{\hat{\theta }}_{1,u}\right)}^{2}+{\left({\hat{\theta }}_{2,t}-{\hat{\theta }}_{2,u}\right)}^{2}}}_{,}\;t,u=1,\ldots ,K$$

The $I_{CG}$ index reported in (19) sums the *weight function* *w* evaluated in every pair of normalized points $({\hat{v}}_{t},{\hat{v}}_{u}),\;t,u=1,\ldots ,K,\;u>t$ and K>1 (the case K=1 must be avoided since at least two images are required to calibrate a two-axis rotary table), then divided by $\sqrt{2}$ times the number of pairs (|E|):

(19) $$I_{CG}=\frac{\sum_{t=1}^{K}\sum_{\begin{array}{c} u=1\\ u>t \end{array}}^{K}{∥{\hat{v}}_{t}-{\hat{v}}_{u}∥}_{2}}{\sqrt{2}\;\left|E\right|}$$

The dividing factor $\sqrt{2}\;\left|E\right|$ is useful for normalizing the index value with respect to the number of chosen images *K*, so that $I_{CG}\in \left[0,1\right]\;\forall K\in N,\;K\;>1$. For instance, the subset in Figure 10a has a $I_{CG}=0.4036$, while the less scattered subset in Figure 10b gives a $I_{CG}$ of 0.2684.

### 5.2. Index-Based Analysis and Experimental Results

Let us now consider the *joint configuration Space* *S*, the *Acquisition set* *A*, the *Calibration set* *C*, the *Test set* *T*, and all the $b_{K}$ subsets $C_{H}\subseteq C$ presented in Section 4, as in Figure 6. Given a $C_{H}$ set of *K* images, the index is

(20) $$I_{CG,H}=\frac{\sum_{t\in H}\sum_{\begin{array}{c} u\in H\\ u>t \end{array}}{∥{\hat{v}}_{t}-{\hat{v}}_{u}∥}_{2}}{\sqrt{2}\;\left|E\right|},\;H\subseteq \left\{3,\ldots ,2r+1,\ldots ,N_{A}\right\},\;r=1,\ldots ,N_{C}$$

**Figure 12:**
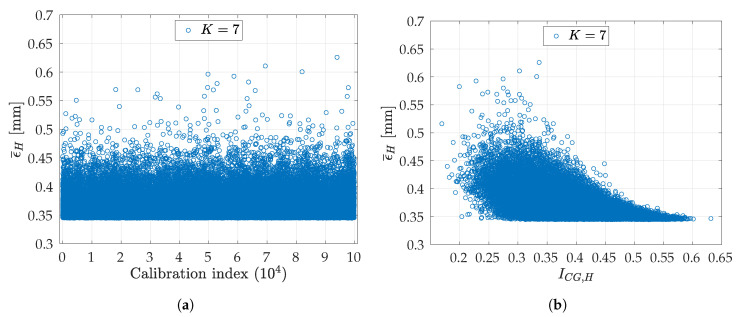
Relationship between ${\bar{\epsilon }}_{H}$ and $I_{CG,H}$ for *N* = 99,885 calibrations made using *N* *C_H_* subsets of *K* = 7 images. These subsets were uniformly downsampled by a factor of Δ = 1000 among all the *b*_7_ combinations of *C_H_*, then finally used for estimating $\left(\omega_{1},\omega_{2},q\right)$, thus obtaining *N* ${\left({\tilde{\omega }}_{1},{\tilde{\omega }}_{2},\tilde{q}\right)}_{H}$: (**a**) the order is the same as in $I_{b_{7}}$; (**b**) the order is the same as in $\bar{I_{b_{7}}}$.

In order to examine the relationship between ${\bar{\epsilon }}_{H}$ and $I_{CG,H}$, K=7 has been selected, since it guarantees a relatively low binomial coefficient (Equation (8)), being $b_{7}=(\begin{array}{c} 50\\ 7 \end{array})=99884400$. Firstly, $I_{CG,H}$ is calculated for all the $b_{7}$ possible combinations of $C_{H}$, obtaining the array $I_{b_{7}}$. Then, this is sorted in ascending order of $I_{CG,H}$, obtaining the ordered array $\bar{I_{b_{7}}}$. In order to reduce the computing time, $\bar{I_{b_{7}}}$ was downsampled by a factor of Δ=1000, obtaining $N=\left\lceil \frac{b_{7}}{\Delta }\right\rceil =99885$ calibrations ${\left({\tilde{\omega }}_{1},{\tilde{\omega }}_{2},\tilde{q}\right)}_{H}$, equally spaced between $\min\bar{I_{b_{7}}}$ and $\max\bar{I_{b_{7}}}$. The corresponding *T-averaged point-to-point errors* ${\bar{\epsilon }}_{H}$ were computed, as expressed in Equation (13), and shown in Figure 12a for all the *N* unsorted calibrations. Finally, these errors are graphed in function of $I_{CG,H}$. Such plot is shown in Figure 12b: increasing the index, the errors tend to decrease. Moreover, it is clear that the greater the value of $I_{CG,H}$, the lower the expected value of ${\bar{\epsilon }}_{H}$ and its variance.-

The same analysis was conducted for K=2, K=4, and K=46 to assess whether the parameter $I_{CG,H}$ was reliable regardless the value of *K*. At first, $I_{CG,H}$ was computed and used for ordering all the $b_{4}$, $b_{46}$, and $b_{2}$ combinations, respectively. Then, at every *K*, the *N* $C_{H}$ subsets were selected by uniformly downsampling by a factor—summed and rounded to the nearest integer index—of $\Delta =\frac{b_{4}}{N}$, $\frac{b_{46}}{N}$, and 1 among all the $b_{4}$, $b_{46}$, and $b_{2}$ combinations of $C_{H}$, respectively. Finally, each combination of images was used for estimating $\left(\omega_{1},\omega_{2},q\right)$, thus obtaining *N* ${\left({\tilde{\omega }}_{1},{\tilde{\omega }}_{2},\tilde{q}\right)}_{H}$.

**Figure 13:**
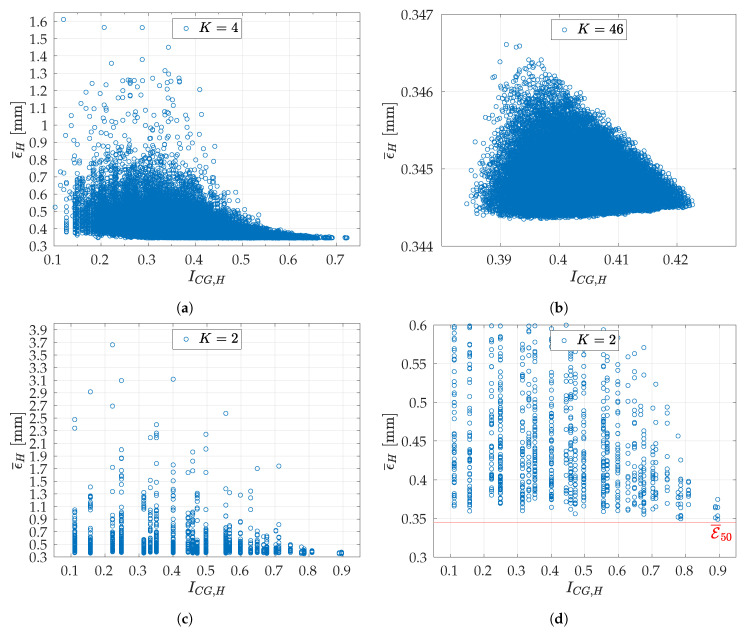
Relationship between ${\bar{\epsilon }}_{H}$ and $I_{CG,H}$ for *N* calibrations and for various *K*: (**a**) *K* = 4 and *N* = 99,885; (**b**) *K* = 46 and *N* = 99,885; (**c**) *K* = 2 and *N* = *b*_2_ = 1225; (**d**) same results as in (**c**), but zoomed-in at the error range [0.3, 0.6] mm.

With reference to Figure 13, it is possible to observe that the trend of ${\bar{\epsilon }}_{H}$, for K=2,4,46, was the same as for K=7 (Figure 12b), provided that the results were sorted in ascending order of $I_{CG,H}$. It is worth noting that the higher the value of *K*, the more flattened the error range. In fact, while for K=2 the errors ranged from around 0.3 mm to 3.7 mm, for K=46 they were between 0.344 mm and 0.346 mm (as could be predicted considering that ${\bar{\sigma }}_{2}\gg {\bar{\sigma }}_{46}$). Additionally, Figure 13 confirms the effectiveness of $I_{CG,H}$ as a method to find the sets which yielded better calibration results, i.e., lower calibration errors. Moreover, considering as a reference the ${\bar{E}}_{K}$ obtained by using all the $N_{C}$ images, i.e., ${\bar{E}}_{50}=0.3446$ mm (Figure 9), and given a subset *H*, it is possible to define the percent relative error as

(21) $$\eta_{H}=\left|\frac{{\bar{E}}_{50}-{\bar{\epsilon }}_{H}}{{\bar{E}}_{50}}\right|\;\cdot \;100\%$$

It is worth noting that, if the *H* corresponding to the highest $I_{CG,H}$ was considered, $\eta_{H}$ was noticeably low for all the tested *K*, as shown in Table 2. As expected, the higher *K*, the lower $\eta_{H}$. However, $\eta_{H}$ was lower than 10% also for K=2. In particular, for K=2, there were four subsets corresponding to the highest $I_{CG,H}$: for the worst $\eta_{H}=$ 8.72%, whereas for the best $\eta_{H}=$ 1.28%. Hence, high-quality calibration results can be achieved even with K=2, provided that the proposed index $I_{CG,H}$ is used to choose the calibration set.

It is reasonable to use the index as a criterion for selecting the angular positions $(\theta_{1}$, $\theta_{2}{)}_{h}$ within the *joint configuration Space* *S*, that is the *K* placements of the checkerboard. The main advantage is that it is able to predict whether a set is going to achieve an effective calibration before acquiring the images. An additional benefit is that it is less time-consuming for a computer, compared to an entire calibration. For instance, the code was tested using a computer equipped with Intel^®^ i7-6700HQ CPU (Dell Inc., Round Rock, TX, USA, 4 physical cores at 2.60 GHz and 8 logical cores), and 32 GB of RAM, then running Matlab^®^ R2020a (The MathWorks Inc., Natick, MA, USA) with parallel pool active on Microsoft Windows^®^ 10. It was observed that the time required to calculate $b_{7}$ times the index $I_{CG,H}$ was about 10 times shorter than the time needed for *N* calibrations. Thus, it is possible to assume that computing the index is 10·Δ=10000 times faster than repeating the calibration for all the $b_{7}$ possible configurations.

## 6. Conclusions

This paper presents a novel approach for the automatic calibration of two-axis rotary tables through optical stereo systems. The proposed optimization algorithm allows for simultaneously calibrating both axes by imaging and processing a checkerboard attached to the rotary table in different poses. A test set of images was defined, which were not exploited during calibration, to assess the quality of the achieved calibration parameters by computing an average reprojection error and its RMS. Moreover, this paper investigated the effect of the number of calibration images on the results. It was shown that the higher the number of images, the lower the achieved mean error and RMS, if the calibration poses are randomly selected. Nevertheless, it was demonstrated that a proper choice of calibration poses can provide high-quality calibration results even with a limited number of images (thus, a shorter calibration time). An index based on the weighted complete graph, $I_{CG}$, was defined, demonstrating that higher values of $I_{CG}$ (i.e., wider dispersion of the calibration poses) correspond to better calibration results. Thus, once the *joint configuration Space* *S* is defined, and given the number of calibration images to be used, it is possible to determine the ideal combination of poses by looking for the highest value of $I_{CG}$. The experimental measurements demonstrated that, using this strategy, the worst calibration error achievable with only two calibration images is marginally higher, about 8%, than the best calibration error achievable with 50 images, thus confirming the advantages of the proposed approach.

## Figures and Tables

**Table 1:** Comparison between the proposed method and the circles algorithm.

Combination	Circles method		Proposed method	
*H*	${\bar{\epsilon }}_{H}$ [mm]	${\bar{\sigma }}_{H}$ [mm]	${\bar{\epsilon }}_{H}$ [mm]	${\bar{\sigma }}_{H}$ [mm]
{59,79,99,93,95,97}	1.6956	0.1488	0.3579	0.1758
{35,55,75,95,93,97,99}	0.8038	0.2310	0.3414	0.1711
{13,33,53,73,93,95,97,99}	0.5283	0.1833	0.3352	0.1678

**Table 2:** $\eta_{H}$ at different *H* and at various *K*.

*K*	$I_{\mathrm{CG},H}$	${\bar{\epsilon }}_{H}\left[\mathrm{mm}\right]$	$\eta_{H}$[%]
2	0.8958	0.3747	8.72
		0.3643	5.73
		0.3533	2.52
		0.3490	1.28
4	0.7225	0.3469	0.67
7	0.6313	0.3466	0.58
46	0.4226	0.3445	0.02

**Figure A1:**
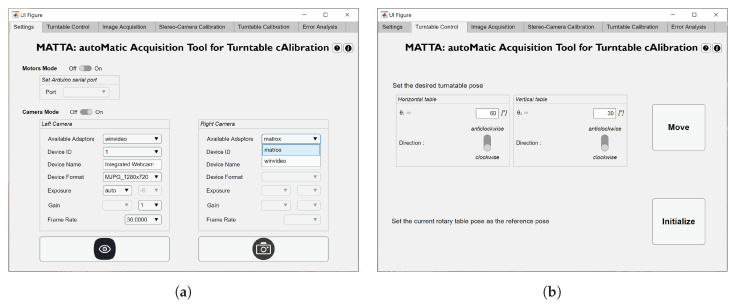
(**a**) *Settings* tab; (**b**) *Turntable Control* tab.

**Figure A2:**
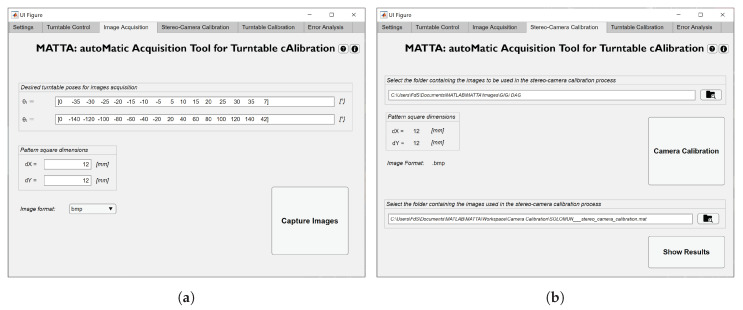
(**a**) *Image Acquisition* tab; (**b**) *Stereo-Camera Calibration* tab.

**Figure A3:**
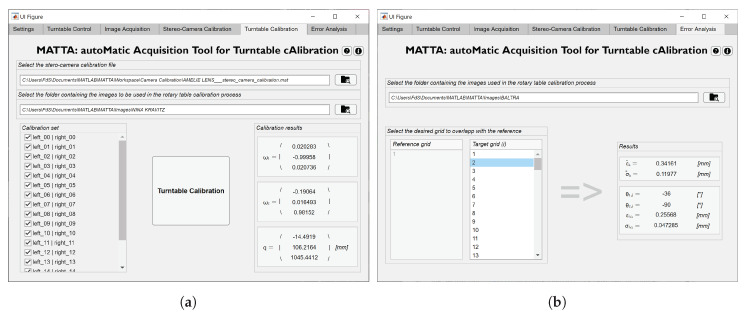
(**a**) *Turntable Calibration* tab; (**b**) *Error Analysis* tab.

**Figure A4:**
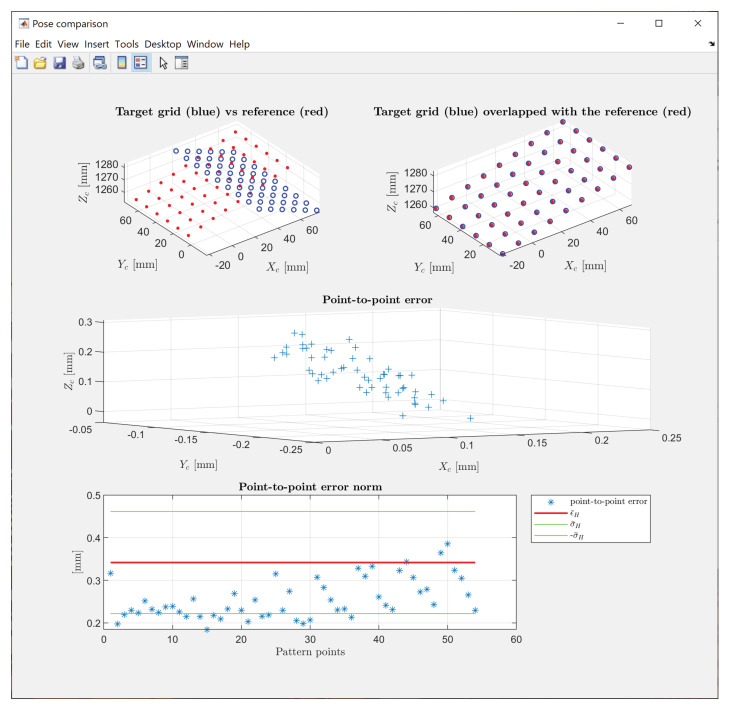
A typical analysis figure displayed once a target grid (in this example, i=2) is selected in the *Error analysis* tab of Figure A3b, showing the following: at the top, the reference grid (${\bar{X}}^{1}$, in red) and the target grid (in blue) before (${\bar{X}}^{2}$) and after (the reprojected grid $X^{1}$) the overlapping, respectively; at the center, their *point-to-point* errors ($\epsilon_{j,i,H},j=1,\ldots ,54\;,\;i=2$, and for a certain *H*); at the bottom, the norm of these errors ($|\epsilon_{j,i,H}|$, blue asterisks), the *T-averaged point-to-point error* (${\bar{\epsilon }}_{H}$, red line) and its standard deviation ($\pm {\bar{\sigma }}_{H}$, green lines). A ${\left({\tilde{\omega }}_{1},{\tilde{\omega }}_{2},\tilde{q}\right)}_{H}$ previously obtained from the *Turntable Calibration* tab was used.

## Appendix A

In this appendix, a brief description of the Graphical User Interface—MATTA: autoMatic Acquisition Tool for Turntable cAlibration—downloadable at [30], is given. The main purpose of the software is to provide a tool that guides the user through the entire calibration process. The software has been developed in the Matlab^®^ R2020a environment [31] and is meant to work on computers equipped with Microsoft Windows^®^ 10, macOS^®^, or Linux^®^. The Computer Vision Toolbox, The Image Processing Toolbox, the Optimization Toolbox, and the Matlab^®^ Support Package for Arduino Hardware are required. The GUI also makes use of the open source Camera Calibration Toolbox for Matlab^®^ provided by the California Institute of Technology [32]. The additional hardware required is described in Section 2. The GUI consists of six tabs:

1. *Settings*;
2. *Turntable Control*;
3. *Image Acquisition*;
4. *Stereo-Camera Calibration*;
5. *Turntable Calibration*;
6. *Error Analysis*.

The *Settings* tab, shown in Figure A1a, allows the user to manage several hardware settings. In more detail, it is possible to

- activate/deactivate the motors connected to the shield via Arduino and to specify its serial port;
- activate/deactivate the camera(s) connected to the computer, adjusting several parameters such as exposure, image format, image resolution, and frame rate.

Moreover, it is possible to test the stereo-camera system configuration performing an image preview or taking a one-shot acquisition.

The second tab, *Turntable Control*, is reported in Figure A1b. It allows the user to rotate the table within the working space in a target configuration determined by the pair of angular positions $\left(\theta_{1},\theta_{2}\right)$. The directions of rotation are according to the rotary table model presented in Section 3, Figure 2. After reaching the desired target configuration, it is possible to set the current table pose as the reference pose ${\bar{X}}^{r}$ for the image acquisition task by means of the *Initialize* button.

The *Image Acquisition* tab, shown in Figure A2a, allows the user to automatically capture the stereo-pair images within the *joint configuration Space* *S* needed for the calibration of the stereo-camera system or of the rotary table. As inputs, the user has to provide the following:

1. the square dimensions of the checkerboard;
2. the desired image format;
3. the target poses of the turntable, in the form of pairs of angular positions ${\left(\theta_{1},\theta_{2}\right)}_{i}$, $i=1,\ldots ,N_{A}$, where $N_{A}$ is the number of acquisitions, with respect to the reference pose ${\left(\theta_{1},\theta_{2}\right)}_{1}=\left(0{}^{{}^{\circ}},0{}^{{}^{\circ}}\right)$.

When the process is completed, the tab automatically creates a folder containing both the stereo-pair images and the associated input variables. It is worth mentioning that the first configuration to be acquired has to be the reference pose.

Subsequently, the *Stereo-Camera Calibration* tab enables the user to properly calibrate the stereo-camera system. With reference to Figure A2b, the user has to specify, as input, the path of a folder of images previously generated with the *Image Acquisition* tab. The software automatically recognizes the key variables and calibrates the stereo-camera system according to the Camera Calibration Toolbox for Matlab^®^. Thus, a stereo-camera calibration file is generated in the *Camera Calibration* folder (inside the *Workspace* folder) labeled with reference to the folder of images deployed in the task. Eventually, it is possible to obtain insights of a stereo-camera calibration file such as intrinsic and extrinsic parameters and geometrical displacement of both the $N_{A}$ grids and cameras in the 3D space by means of the *Show Results* button.

Furthermore, the user is able to calibrate the two-axis rotary table by means of the *Turntable Calibration* tab, shown in Figure A3a. As inputs the user has to provide the following:

1. a stereo-camera calibration file previously generated in the *Stereo-Camera Calibration* tab;
2. a folder of images previously acquired with the aid of the *Image Acquisition* tab. The GUI fills the *Calibration set* panel with a number of checkboxes equal to the number of stereo-pair images contained in the specified folder, i.e., $N_{A}$. By means of the checkboxes, the user is able to select the *K* stereo-pair images to be used in the calibration procedure, thus constituting the desired subset *H*; alternatively, the entire set of $N_{A}$ images is used by default.

The rotary table calibration method is according to the one presented in Section 3. At the end of the process, a table calibration file is generated, and the estimated values of the unit vectors $\omega_{1}$ and $\omega_{2}$, as well as the intersection point *q*, i.e., ${\left({\tilde{\omega }}_{1},{\tilde{\omega }}_{2},\tilde{q}\right)}_{H}$, are displayed in the tab.

The last tab offers an in-depth analysis of the errors committed during a turntable calibration. As input, only a folder of $N_{A}$ images—of which *K* are previously used for calibrating the rotary table—must be provided. At first, the computed *T-averaged point-to-point error* ${\bar{\epsilon }}_{H}$, as well as its standard deviation ${\bar{\sigma }}_{H}$, are displayed. Then, as Figure A3b shows, the *Error Analysis* tab fills the *Target grid* panel with ${\bar{X}}^{i}$, $i=1,\ldots ,N_{A}$, Target grids. Finally, once a desired target grid has been selected by the user, a summarizing figure is displayed, as shown in Figure A4.

It is worthwhile to mention that, for each tab, various errors are properly treated by MATTA, as well as warnings, and that numerous informative messages stating the current status of the running process are displayed.
